# Evaluation of the apical sealing of an eggshell hydroxyapatite-based sealer 

**DOI:** 10.4317/jced.60743

**Published:** 2023-11-01

**Authors:** Carlos-Eduardo Fontana, Beatriz-Anjos dos Santos, Mônica-Celes-Nascimento-Machado Campos, Samara-Gonçalves-Félix de Lima, Venâncio-Castro da Silva, Aline-Dentini-Luque Gonçalves, João-Daniel-Mendonça de Moura, Daniel-Guimarães-Pedro Rocha, Sérgio-Luiz Pinheiro, Carlos-Eduardo-da Silveira Bueno

**Affiliations:** 1 Pontifical Catholic University of Campinas (PUC-Campinas), Center for Health Sciences, Postgraduate Program in Health Sciences, Campinas, São Paulo, Brazil; 2 Undergraduate Dentistry and Scientific Initiation PUC-Campinas, Center for Health Sciences, Campinas, São Paulo, Brazil; 3 Department of Endodontics, Federal University of Pará, Belém, Pará, Brazil; 4 Department of Endodontics, PUC-Campinas, Center for Health Sciences, Campinas, São Paulo, Brazil; 5 Department of Endodontics, Faculdade São Leopoldo Mandic, Instituto de Pesquisas São Leopoldo Mandic, Campinas, SP, Brazil

## Abstract

**Background:**

The success of endodontic treatment can be influenced by the type of endodontic sealer used, as certain sealers may be prone to apical microleakage, leading to treatment failure. The limitations of currently available sealers necessitate the development of new materials to improve the success rate of endodontic treatment. Therefore, the objective of this study was to assess the apical microleakage of newly developed hydroxyapatite-based endodontic sealers, including one derived from eggshells, and compare them with other commercially available sealers.

**Material and Methods:**

Eighty-five extracted human upper anterior teeth were selected for this study. The teeth were divided into 5 experimental groups and 2 control groups. The experimental groups were designated as follows: (1) HPSINT - obturated with gutta-percha cone and synthetic hydroxyapatite-based sealer, (2) BIOC - obturated with gutta-percha cone and Bio C-Sealer sealer, (3) AHPLUS-BC - obturated with gutta-percha cone and AHPLUS Bioceramic sealer, (4) AHP - obturated with gutta-percha cone and AHPLUS sealer, and (5) HPO - obturated with gutta-percha cone and sealer based on hydroxyapatite extracted from eggshells. Additionally, there were positive and negative control groups consisting of instrumented teeth filled with gutta-percha cones without any sealer and instrumented teeth without any filling, respectively. Methylene blue dye penetration was used to assess apical microleakage. Descriptive statistical analysis and Shapiro-Wilk normality test were applied to the observed results. As the samples followed a normal distribution, the ANOVA test was applied.

**Results:**

The control groups confirmed the validity of the experimental method, while the experimental groups showed varying degrees of dye penetration. The group obturated with Bio C-Sealer exhibited the highest mean apical microleakage, while AHPLUS Bioceramic sealer demonstrated lower mean than AHPLUS sealer and sealer based on hydroxyapatite extracted from eggshells (*p*<0.05). Finally, there was no difference between the synthetic hydroxyapatite-based sealer and AHPLUS Bioceramic sealer, AHPLUS sealer and sealer based on hydroxyapatite extracted from eggshells (*p*>0.05). No significant difference was observed between the hydroxyapatite-based sealers and the AHPLUS-BC sealer.

**Conclusions:**

The results of this study suggest that the newly developed hydroxyapatite-based endodontic sealers, including the one derived from eggshells, may have a lower risk of apical microleakage compared to other commercially available sealers. These findings highlight the potential of hydroxyapatite-based sealers to improve the success rate of endodontic treatment. Further research and clinical studies are warranted to validate these results and explore the long-term effects of these novel sealers.

** Key words:**Endodontic treatment, apical microleakage, endodontic sealer, hydroxyapatite, eggshell-derived sealer.

## Introduction

Endodontic treatment involves the diagnosis, prevention, and treatment of diseases or injuries to the dental pulp and surrounding tissues. Endodontic treatment typically involves several stages, including access preparation, cleaning, and shaping the root canal system, and obturation or filling of the canal. Anatomical challenges can affect each stage of endodontic treatment and may require special techniques or materials to overcome. (1). The type of endodontic sealer used to fill the root canal can affect the success of endodontic treatment. One reason for this is that certain types of sealers have a greater tendency to experience apical microleakage, which can lead to treatment failure (2).

Apical microleakage occurs when bacteria or other contaminants penetrate the root canal through a gap between the filling material and the tooth structure, usually at the tip of the root. This can cause inflammation and infection in the periapical tissues, leading to pain, swelling, and potentially even tooth loss (3). Factors that can contribute to apical microleakage include the type of sealer used, its handling properties, the quality of the root canal preparation and filling, and the presence of any cracks or defects in the tooth structure. Some types of endodontic sealers are more susceptible to contraction during setting, which can cause gaps to form between the filling material and the tooth surface (4).

Currently available materials for filling root canals have improved significantly over time, but they still have certain weaknesses that can justify the need for the development of new endodontic sealers (5). Many currently available sealers have the inability to bond adequately to dentin, leading to the formation of gaps and subsequent failure of the filling material. Furthermore, some endodontic sealers may have adverse effects on the periapical tissues or cause allergic reactions in patients, indicating the need for biocompatible materials that can be used safely in a clinical setting (6). Therefore, the limitations of currently available endodontic sealers justify the need for the development of new and improved materials to address these weaknesses and improve the overall success rate of endodontic treatment.

Hydroxyapatite is the natural mineral constituent found in bone representing from 30 to 70% of the mass of bones and teeth. The synthetic hydroxyapatite has biocompatibility and osseointegration properties, which makes it a substitute of human bone in implants and prostheses (7,8), hence the great interest in its production. These properties added to its high ability to adsorb and/or absorb molecules make hydroxyapatite an excellent support for prolonged action of anticancer drugs in the treatment of tumors bones, and also efficient in the treatment of removal of heavy metals in water and polluted soils (7,8). The development of granulated hydroxyapatite is of great interest due to its wide use in the area orthopedics and traumatology (medicine and dentistry). The hydroxyapatite synthetic is biocompatible, it is not carcinogenic nor allergenic, so it is a safe and clinically accepTable material.

Hydroxyapatite is a mineral that plays a crucial role in the formation and maintenance of teeth and bones. It is composed of calcium phosphate and has a unique crystal structure that gives it exceptional strength and hardness. While hydroxyapatite can be synthesized artificially in a laboratory, it can also be found in natural sources such as the tooth and eggshell. In particular, the use of hydroxyapatite extracted from eggshells or from human teeth as a filler in endodontic sealers may show potential due to its low cost, biocompatibility, and possibility to improve the mechanical properties of the sealer, making it a promising material for use in endodontic sealers (9). This has the potential to revolutionize the field of dentistry by providing a biocompatible, low-cost, and effective endodontic sealer.

Therefore, the use of hydroxyapatite extracted from eggshells in the creation of new endodontic sealers could help overcome some of the weaknesses of currently available materials and improve the overall success rate of endodontic treatment. Based on that, the aim of this study was to assess whether newly developed hydroxyapatite-based endodontic sealers could have a smaller apical microleakage than the sealers already used in the endodontic treatment. The null hypothesis is that there is no significant difference in apical microleakage between the newly developed hydroxyapatite-based endodontic sealers, and the commercially available sealers currently used in endodontic treatment.

## Material and Methods

Eigthy-five permanent upper anterior teeth donated by patients at the Clinic of the School of Dentistry of Pontifical Catholic University of Campinas (PUC-Campinas) with the signing of an Informed Consent Form were selected. Due to this fact, the study was submitted and approved by the institution’s Ethics and Research Committee number 3.653.397. The teeth used in the research were extracted for various reasons, such as lack of masticatory function, teeth with caries lesions that could not be restored, infraoccluded teeth, malpositioned and extracted teeth, therefore, with clear clinical indication for extraction.

The inclusion criteria for the sample were defined as: complete root formation and uncalcified canals, absence of previous endodontic treatment, absence of pathological external and/or internal root resorption, absence of cracks or root fractures, root length equal to or greater than 15mm, canals with patent foramens, and teeth with straight roots and no degree of curvature (10). On the other hand, the exclusion criteria included incomplete root formation, presence of previous endodontic treatment, teeth with cracks or root fractures, teeth with obstructed/calcified canals, teeth with pathological internal and/or external root resorption, and teeth with root angulation (10).

After obtaining teeth that met the previously listed criteria, the root surfaces were gently scraped with periodontal curettes No. 13 and 14 (Hu-Friedy, Chicago, USA) to remove any remnants of periodontal ligament and adherent tartar, followed by smoothing with Robson brushes (MKlife produtos medical e dental LTDA, Porto Alegre, Brazil). Subsequently, ultrasonic cleaning was performed, and the teeth were conditioned in 0.1% thymol for 24 hours for disinfection. Once this step was completed, the teeth were rinsed and stored in distilled water until the time of the study, pending laboratory clearance for use (2).

The teeth were previously analyzed using an Operating Microscope (DFVasconcellos, Rio de Janeiro-RJ, Brazil) at a magnification of 25X to observe possible apical resorptions or anatomical abnormalities that could hinder the complete instrumentation of the teeth’s canals. In addition, teeth that showed any signs of external root cracks were excluded from the study.

For the beginning of the experiment, the crowns of the teeth were sectioned at the level of the cemento-enamel junction (CEJ) using a diamond bur 2135 (American Burrs, Palhoça, Brazil) at high rotation, obtaining roots of approximately 15 mm measured with a digital caliper Series 500 DIN 862 (Mitutoyo, São Paulo-SP, Brazil). This allows for better standardization of the total length of the roots and, therefore, the canals, with the objective of a linear evaluation of apical leakage equated among the samples.

All teeth were instrumented with the WaveOne Gold Large System (11) (Dentsply Maillerfer, Bllaiguess, Swiss). The instrumentation was performed by a single operator using the X-Smart Plus motor (Dentsply Maillerfer, Bllaiguess, Swiss) in WAVE ONE GOLD reciprocating mode. The speed (RPM) and torque (N) settings were followed according to the parameters indicated by the manufacturer of the respective instrument.

Root canal instrumentation was carried out at the working length, which was determined to be 1mm short of the apical foramen. This reference point was obtained in each canal when the K-file #10 (Dentsply Maillerfer, Bllaiguess, Swiss) visually reached the foramen, and 1mm was subtracted from this distance (11). Apical observation was facilitated using an operating microscope at 25X magnification.

During instrumentation, a K-file #10 (Dentsply Maillerfer, Bllaiguess, Swiss) was used for foraminal patency after each sequence of radicular third preparation. The irrigating solution used during preparation was 1% sodium hypochlorite (2), applied in a quantity of 1ml for each instrument change.

At the end, the same irrigation protocol was used for all teeth, consisting of 3 cycles of 20 seconds of ultrasonic agitation with the E1 insert at 20% power (Helse, São Paulo, Brazil) per canal, using 3ml of 17% EDTA, followed by another repetition of 3 cycles of 20 seconds with the irrigant used during instrumentation (12).

A total of 85 teeth were randomly distributed into 5 experimental groups of 15 teeth each and 2 control groups of 5 teeth each. For this purpose, a specific randomization program (http://www.random.org) was used. The division into experimental groups corresponded to the type of endodontic obturation that would be performed subsequently:

• HPSINT (1) - 15 teeth obturated with gutta-percha cone and synthetic hydroxyapatite-based sealer;

• BIOC (2) - 15 teeth obturated with gutta-percha cone and Bio C-Sealer sealer (Angelus, Londrina, Brazil);

• AHPLUS-BC (3) - 15 teeth obturated with gutta-percha cone and AHPLUS Bioceramic sealer (Dentsply Maillerfer, Bllaiguess, Swiss);

• AHP(4) - 15 teeth obturated with gutta-percha cone and AHPLUS sealer (Dentsply Maillerfer, Bllaiguess, Swiss);

• HPO(5) - 15 teeth obturated with gutta-percha cone and sealer based on hydroxyapatite extracted from eggshells.

• Positive Control - In this group, five teeth were instrumented and filled with gutta-percha cones without the use of any sealer. This control group serves as a reference point to assess the apical sealing ability solely based on the gutta-percha filling without the influence of any sealing material.

• Negative Control - 05 teeth only instrumented without any filling with sealer or gutta-percha cone.

The hydroxyapatite powder extraction process began chicken eggshells. The eggshells were crushed, dehydrated, and washed in a hydroalcoholic solution. Afterward, the shells were dried. The dehydrated shells were finely triturated into a uniform, yellowish powder.

The obtained calcium oxide was subjected to thermal treatment in two cycles of calcination in a muffle furnace, resulting in a transformation from calcium carbonate to calcium oxide. The hydroxyapatite synthesis involved a controlled reaction between calcium oxide and phosphoric acid. The acid was added to the calcium oxide solution using controlled temperature and pH conditions.

The resulting solution underwent a transformation from liquid to viscous during acid addition, necessitating the addition of water and further acid. The pH was frequently adjusted using ammonia. After the synthesis, the solution was decanted, and then the hydroxyapatite powder was collected through siphoning. The collected hydroxyapatite was dried in an oven and then subjected to calcination.

Infrared analysis confirmed the formation of hydroxyapatite in the obtained powder, demonstrating similar peaks to standard hydroxyapatite and even human dental hydroxyapatite.

To obtain the obturating sealer, a portion of hydroxyapatite powder extracted from eggshells was obtained using the powder dispenser of the Ketac Molar Easymix glass ionomer cement (3M, Deutschland, Germany). Three drops of the polyacrylic acid from Ketac Molar Easymix (3M, Deutschland, Germany) were dispensed, and the mixture was spatulated for approximately one minute.

Initially, each tooth in its respective group was assigned a number and identification letter. All teeth were obturated with single-cone gutta-percha and main cone with a size of 45 and taper of .05 (Dentsply Maillerfer, Bllaiguess, Swiss) equivalent to the apical preparation of the instrumentation. Endodontic sealers were spatulated until fully homogenized and inserted into the root canal with the aid of a Centrix syringe (Maquira, Maringa, Brazil) and a needle tip. Subsequently, the gutta-percha cone (Dentsply Maillerfer, Bllaiguess, Swiss), coated with the same sealer, was introduced into the root canal. Then, a Schilder-compatible condenser, compatible with the cervical diameter of the root canal, was used to remove the excess material, and thus, vertical condensation. Digital periapical radiographs in two directions were taken to analyze the quality of the obturation. 

The root canal orifices were sealed with temporary sealer Cimpat (Septodont, Santa Catarina, Brazil), and the entire root canal length, except for the apical 2mm and the foramens, was sealed with two layers of nail polish. This allowed for all possible areas of future dye penetration into the canal to be sealed, allowing only possible infiltration in the apical region, which would be evaluated later. An exception to the nail polish sealing procedure was performed in the negative control group, where the entire extension, including the 2mm apical portion and the foraminal area, was sealed with nail polish in this case. After drying of the enamel applied on the specimens, they were stored in an oven at 37°C with 100% humidity for seven days for complete setting of the endodontic sealer. After this time, the teeth were then submerged in a solution containing 1% methylene blue for 72 hours.

**Figure 1:**
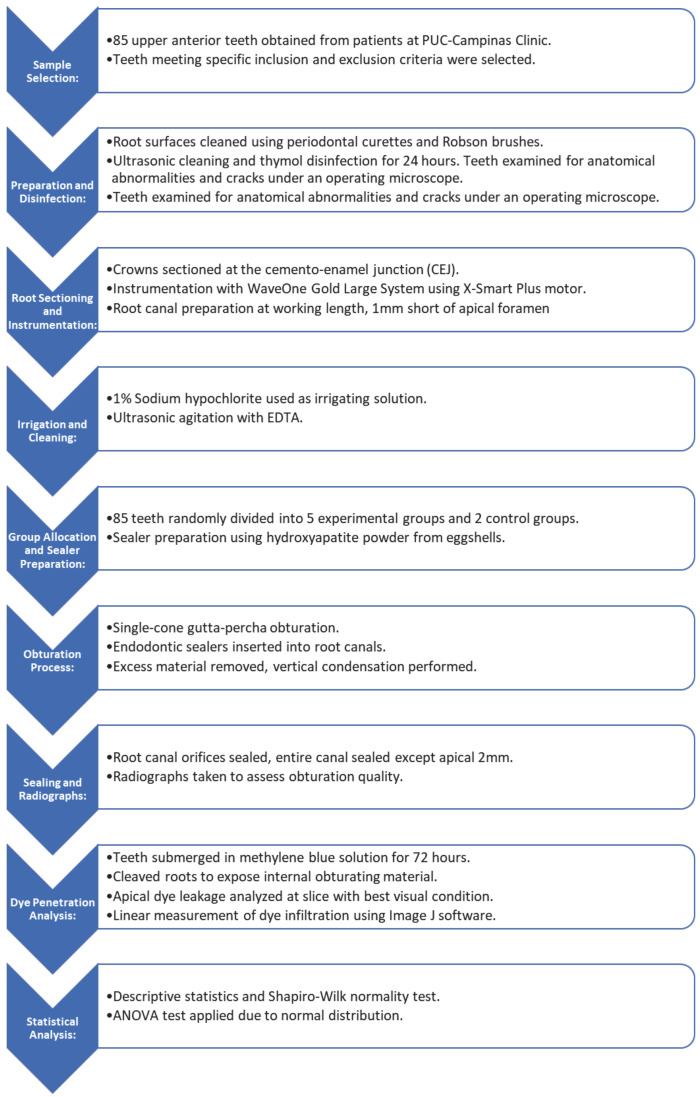
Graphic summary.

After 72 hours of immersion in the methylene blue solution, the teeth were washed and sequentially grooved in the mesio-distal direction using a low-speed metal disc under refrigeration. Subsequently, they were cleaved using a spatula no. 24 (Golgran, São Paulo, Brazil) and forceps no. 1 (Golgran, São Paulo, Brazil) to expose the internal obturating material. With the cleaving of the roots, the apical dye leakage analysis was applied to the slice with the best visual condition for linear measurement of dye infiltration. Photos were taken using an operative microscope at 12.5X magnification. The detailed methodology outlined in this section is succinctly summarized in the provided graphic summary bellow, (Fig. 1).

The Image J software was used by a single experienced operator for linear measurement of dye infiltration in millimeters (mm) at the apical portion of the root, in a cervical direction. The data were tabulated for statistical analysis. Descriptive statistical analysis and Shapiro-Wilk normality test were applied to the observed results. As the samples followed a normal distribution, the ANOVA test was applied.

## Results

The negative control group showed no dye leakage, while the positive control groups exhibited complete infiltration throughout the canal space, thus confirming and validating the experimental method.

**Figure 2:**
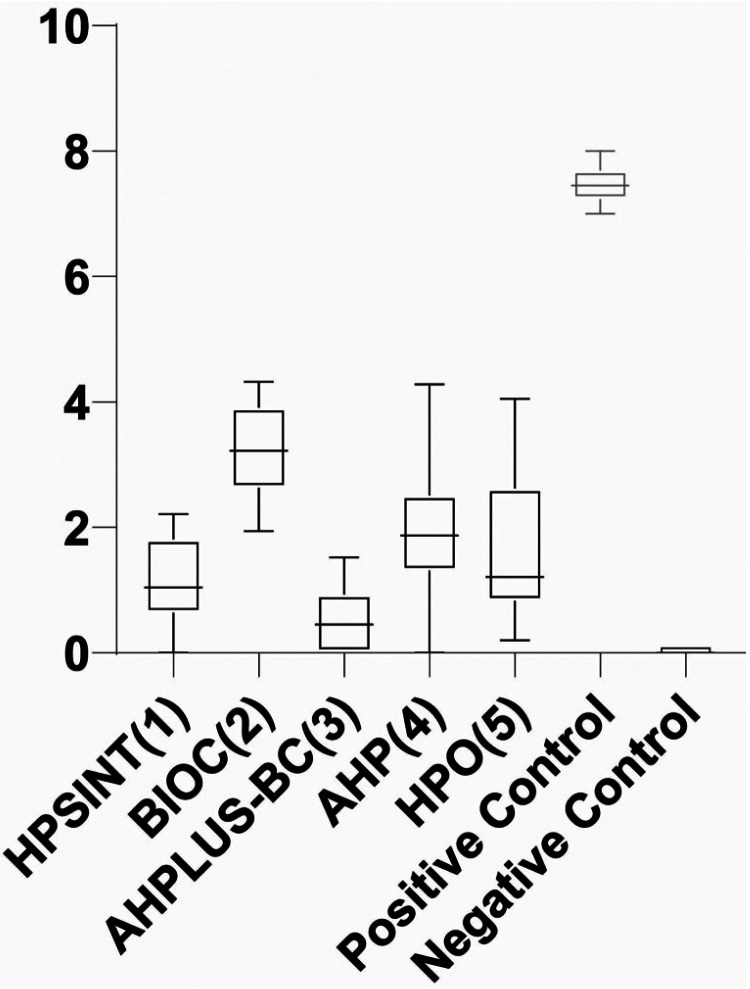
Graphical representation of the obtained results.

**Figure 3:**
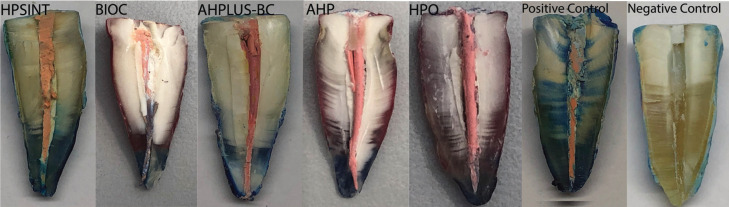
Photographs of a sample of each group representing the results of the work.

BIOC(2) has shown the highest mean of apical microleakage between all investigated sealers (*p*<0.05). AHPLUS-BC(3) demonstrated lower mean than AHP(4) and HPO(5) (*p*<0.05). Finally, there was no difference between the HPSINT(1) and AHPLUS-BC(3), AHP(4) and HPO(5) (*p*>0.05). All the results are demonstrated in Table 1 and Figure 2. Figure 3 is a representative image of the results found in the study.

**Table 1:**
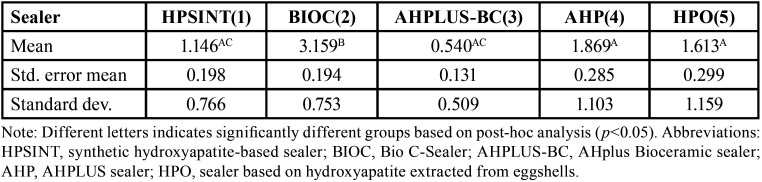
Comparison of means and standard deviations (in millimeters) for different sealers in terms of apical microleakage.

## Discussion

The methodology employed in this study was straightforward, yet it allowed for interesting findings to be revealed, particularly in the case of the HPO(5) group. The results showed that BIOC(2) had the highest mean of apical microleakage, suggesting potential limitations in its sealing ability, which may be attributed to factors such as lack of moisture or setting time. When evaluating the efficacy of new endodontic materials in the laboratory, it is important to acknowledge a significant limitation regarding the difficulty in maintaining the appropriate moisture level for testing the bioceramic material *in vitro*. In our study, we observed that BIOC(2), a endodontic sealer from Angelus, exhibited inferior results compared to other tested materials. This disparity may be attributed to the fact that the bioceramic sealers relies on moisture derived from the tissue itself, specifically the dentinal tubules, which cannot be fully replicated under laboratory conditions (18). Therefore, it is possible that the outcomes obtained *in vitro* fall short of what is typically observed in *in vivo* studies.

On the other hand, AHPLUS-BC(3) demonstrated a significantly lower mean of microleakage compared to BIOC(2), AHP(4), and HPO(5), indicating a potential advantage in terms of sealing efficacy. Notably, HPSINT(1) and HPO(5) were found to be equivalent to AHP(4), one of the most cited endodontic sealers in the world, which is a positive result. The use of a single experienced operator strengthens the findings by minimizing potential operator-related biases. Furthermore, the findings of this study provide valuable insights into the comparative performance of different sealers in terms of apical microleakage, which may have implications for clinical decision-making in endodontic practice.

Biomaterials are accepted by living tissues and share certain similarities with them. They can be natural or synthetic, and examples of bioceramics, which are widely used in medicine and dentistry as an important class of biomaterials, include hydroxyapatite, alumina, zirconia, and bioactive glasses (13). In nature, calcium is never found in isolation, but rather as the main component of calcareous rocks such as marble (CaCO3), gypsum (CaSO4.2H2O), and fluorite (CaF2). Apatite, a calcium fluorophosphate, is another common calcium mineral, as it can be found in teeth, eggshells, pearls, shells of marine animals, among others (14).

Hydroxyapatite is the major component of bone and exhibits high strength, bioactivity, and biocompatibility (19). Upon analyzing these concepts, it becomes apparent that hydroxyapatite sealer derived from eggshells may exhibit biocompatibility with dental substrates due to their chemical similarity. This perspective is relevant in the context of endodontics, specifically in root canal obturation, which refers to the process of filling the space previously occupied by the pulp after chemical mechanical preparation (15). The quality of canal sealing is crucial for reestablishing and maintaining the health of apical and periapical tissues. Commonly used materials for root canal obturation include gutta-percha and certain types of sealers (4).

The sealers studied exhibited a diverse range of chemical compositions, which may have contributed to the observed differences in their performance. This variance in composition likely played a pivotal role in influencing the results obtained in this study. Eggshell powder, constituting predominantly of calcium carbonate (98.2%) along with trace amounts of magnesium (0.9%) and phosphate (0.9%), is an essential source of calcium (20,21). Bio-C Sealer, on the other hand, comprises a blend of calcium silicates, calcium aluminate, calcium oxide, zirconium oxide, iron oxide, silicon dioxide, and dispersing agents (22). Meanwhile, the components of Ah Plus Paste A and Paste B encompass bisphenol-A epoxy resin, bisphenol-F epoxy resin, calcium tungstate, zirconium oxide, silica, iron oxide pigments, dibenzyldiamine, aminoadamantane, tricyclodecane-diamine, and calcium tungstate, among others. Similarly, AH Plus Bioceramic is formulated with zirconium dioxide, tricalcium silicate, dimethyl sulfoxide, lithium carbonate, and thickening agents (23). These distinct compositions likely contributed to the observed variations in apical microleakage among the sealers.

In order to improve the technique and optimize the materials used in root canal obturation for maximum effectiveness and longer-lasting treatment outcomes, it is necessary to associate these sealers with gutta-percha cones to ensure a three-dimensional hermetic seal of the root canal systems (16,24). In this context, the endodontic market has been actively searching for the development of new sealers that yield more satisfactory results in tests (25). One such sealer that deserves attention is the hydroxyapatite-based sealer Ca10(PO4)6(OH)2, which is a bioceramic known for its high success rates in promoting bone development and strong bonding with the host surface. This biocompatibility is attributed to its chemical similarity with the mineral phase of bones and teeth (17,26).

According to Zhekov and Stefanova (27), bioceramic endodontic sealers are known for their high hydrophilicity, making them well-suited for sealing root perforations and resorptions. These sealers exhibit a range of desirable characteristics, including low toxicity, a high pH, the ability to induce hydroxyapatite formation, activation of regenerative responses, biocompatibility, and bioactivity. When compared to epoxy resin-based sealers, bioceramic sealers not only share similar traits but also offer distinct advantages (28-30). As the quest for optimized endodontic materials and enhanced biocompatibility continues, the utilization of a hydroxyapatite sealer derived from eggshells has garnered attention as a promising alternative (31). This interest arises from the fact that eggshell, a bioceramic compound, exhibits ornal te akin to hydroxyapatite (14).

Polyacrylic acid, from a chemical standpoint, exhibits a proclivity for interacting with the hydroxyapatite chains, particularly with regards to calcium ions (32). This engenders the formation of calcium polyacrylate chains, thereby precipitating a profound engagement. The utilization of polyacrylic acid, therefore, stems from an informed understanding of its potential for effecting chemical polymerization through the said interaction.

The use of hydroxyapatite sealer derived from eggshells can enhance the biocompatibility of the material due to its ornal t similarity with dental structure. Furthermore, it suggests that the bonding of the new material to the dental structure of the same composition may promote better sealing inside the root canals. Linear dye infiltration analysis revealed that both the AHP(4) and HPO(5)-obturated sealers showed similar infiltration ornal in the interior space of the canal. However, upon visual analysis of microscope images, it appears that the positive control group and the AHP(4)-obturated group showed greater infiltration than the HPO(5)-obturated group, although this was not statistically significant compared to the AHP(4) group.

Thus, the formulation of bioceramic sealers from more accessible materials such as eggshells may have a favorable prognosis. Even with numerous formulations available in the market, the clinical application of each case should be taken into consideration, as formulations will have advantages and disadvantages. In any case, orn should ornal t a favorable long-term prognosis.

In this study, we aimed to conduct a ornal te investigation to evaluate the sealing ability of ornal te sealers. This was the first step towards further testing of orna characteristics such as cytotoxicity, antimicrobial effect, radiopacity, and biocompatibility. Our primary focus was to determine whether the tested sealers had adequate sealing capabilities. The results obtained in this study provide valuable insights into the performance of ornal te sealers in terms of apical microleakage. However, further testing is necessary to fully assess the potential of these sealers. In future studies, we plan to investigate orna ornal te characteristics of these sealers to provide a more comprehensive understanding of their clinical utility.

Furthermore, it is ornal te to consider that the formulation and manufacture of such sealer would not entail high costs, making it suiTable for use in services provided by the public health system, as orn do not require extensive resources. Moreover, there is the possibility of reducing environmental impact by reusing eggshells, resulting in a clinically effective and environmentally friendly product. Therefore, after discussing these concepts and conducting tests, it can be understood that hydroxyapatite sealer derived from eggshells may be promising in terms of compatibility with dental tissues and sealing of the root canal system, as evidenced by the good sealing observed in the initial infiltration test.

## Conclusions

Overall, this study contributes to the existing literature by providing insights into the sealing efficacy of ornal te endodontic sealers and highlighting observed ornal te among them. The conducted tests demonstrate the promising potential of hydroxyapatite sealer derived from eggshells. However, further research in this orn may be necessary to confirm these findings and investigate the underlying mechanisms, with the ultimate aim of enhancing the success and longevity of endodontic treatments.
